# Loss of p16^Ink4a^ Function Rescues Cellular Senescence Induced by Telomere Dysfunction

**DOI:** 10.3390/ijms13055866

**Published:** 2012-05-16

**Authors:** Xiufeng Zhang, Xiaoming Wu, Wenru Tang, Ying Luo

**Affiliations:** 1 Faculty of Environmental Science and Engineering, Kunming University of Science & Technology, Kunming 650224, China; E-Mails: xiuzheng1203@163.com (X.Z.); xiao_tracy@163.com (X.W.); 2 Lab of Molecular Genetics of Aging & Tumor, Faculty of Life Science and Technology, Kunming University of Science & Technology, Kunming 650224, China

**Keywords:** p16^Ink4a^, Werner Syndrome, telomere dysfunction

## Abstract

p16^Ink4a^ is a tumor suppressor and a marker for cellular senescence. Previous studies have shown that p16^Ink4a^ plays an important role in the response to DNA damage signals caused by telomere dysfunction. In this study, we crossed *Wrn^−/−^* and *p16^Ink4a−/−^* mice to knock out the *p16^Ink4a^* function in a Wrn null background. Growth curves showed that loss of p16^Ink4a^ could rescue the growth barriers that are observed in *Wrn^−/−^* mouse embryonic fibroblasts (MEFs). By challenging the MEFs with the global genotoxin doxorubicin, we showed that loss of p16^Ink4a^ did not dramatically affect the global DNA damage response of *Wrn^−/−^* MEFs induced by doxorubicin. However, in response to telomere dysfunction initiated by the telomere damaging protein TRF2^ΔBΔM^, loss of p16^Ink4a^ could partially overcome the DNA damage response by disabling p16^Ink4a^ up-regulation and reducing the accumulation of γ-H2AX that is observed in *Wrn^−/−^* MEFs. Furthermore, in response to TRF2^ΔBΔM^ overexpression, *Wrn^−/−^* MEFs senesced within several passages. In contrast, *p16^Ink4a−/−^* and *p16^Ink4a−/−^Wrn^−/−^* MEFs could continuously grow and lose expression of the exogenous TRF2^ΔBΔM^ in their late passages. In summary, our data suggest that in the context of telomere dysfunction, loss of p16^Ink4a^ function could prevent cells from senescence. These results shed light on the anti-aging strategy through regulation of p16^Ink4a^ expression.

## 1. Introduction

Extensive studies have demonstrated that telomere dysfunction could induce cellular senescence, ultimately resulting in either aging or cancer [1–4]. Telomere dysfunction, including telomere shortening due to a lack of telomerase function, telomere uncapping and structural damage due to defects in proteins maintaining telomere structure, could be recognized as a DNA damage signal that triggers the DNA damage response (DDR) in mammalian cells [5,6]. Depending on the intactness of the DDR system, for example, the status of the ATM-p53-p21 pathway, telomere dysfunction could either prevent or promote tumorigenesis [7–9].

The role of the Rb-p16^Ink4a^ pathway in response to DNA damage induced by telomere dysfunction has been controversial. p16^Ink4a^ regulates cell cycle progression by specifically binding to and inhibiting the cyclin-dependent kinases Cdk4 and Cdk6. It has previously been demonstrated that p16^Ink4a^ accumulates in conjunction with p38 activation in ras-induced senescent cells and that this accumulation is essential for senescence [10]. It has also been shown that p16^Ink4a^ accumulates in both human and mouse cells as a function of replicative senescence [11,12]. In a previous study, telomere dysfunction was induced by inhibiting the telomere binding protein TRF2 with the dominant negative protein TRF2^ΔBΔM^. This study demonstrated that the telomere damage induced by TRF2^ΔBΔM^ induces p16^Ink4a^ accumulation. Inhibition of p16^Ink4a^ with shRNA or overexpression of BMI1 (a p16^Ink4a^ transcription regulator) showed a nearly complete bypass of the telomere-directed senescence in p53-deficient cells [13]. These data demonstrated that in human cells, p16^Ink4a^ plays an important role along with the p53 pathway in the DDR to telomere dysfunction. This same study demonstrated that in wild type MEFs, the loss of p53 function was sufficient to completely abrogate the senescence arrest, indicating that in the absence of p53, the loss of p16^Ink4a^ function is not necessary for mouse cells to bypass senescence [13]. In addition, previous studies have shown that inhibition of TRF2 results in apoptosis in MEFs and that this response is mediated by ATM and p53 [14].

Wrn, a member of the RecQ family of DNA helicases, is deficient in patients with Werner syndrome (WS) [15]. WS is an autosomal recessive progeric disease that is characterized by premature atherosclerosis, ischemic heart disease, osteoporosis, and cataracts [16]. A previous study showed that inhibition of the stress-induced p38 MAP kinase could prevent the senescence of WS cells [17]. Fibroblasts from WS patients have accelerated telomere erosion and senescence that can be rescued by the overexpression of telomerase, suggesting that telomere dysfunction may play an essential role in the manifestation of the WS phenotype [18]. To address this hypothesis, a mouse model of WS with a double knockout of *mTerc* and *Wrn* was generated, which faithfully manifested the human phenotype of WS [19,20]. The Wrn protein is necessary for efficient replication of G-rich telomeric DNA, preventing telomere dysfunction and consequent genomic instability [21]. In addition, the function of Wrn in telomere regulation is regulated by TRF1 and TFR2 [22]. Thus, a mouse model deficient in Wrn function (*Wrn^−/−^*) provides a very useful tool for investigating the factors involved in aging in response to telomere dysfunction.

By crossing mice deficient in p16^Ink4a^ function (*p16^Ink4a−/−^*) with mice deficient in Wrn function (*Wrn^−/−^*), we generated mouse embryonic fibroblasts (MEFs) with the following genotypes: *p16^Ink4a−/−^* only, *Wrn^−/−^* only, and *p16^Ink4a−/−^Wrn^−/−^*. Furthermore, we challenged these cells with either the global genomic DNA damage reagent doxorubicin or the telomere specific damaging protein TRF2^ΔBΔM^. By comparing their growth rates, DDR, senescence status, and genomic instability, we investigated the role of p16^Ink4a^ in the induction of senescence by Wrn-related telomere dysfunction.

## 2. Results and Discussion

### 2.1. Loss of p16^Ink4a^ Function Overcame the Growth Barrier Caused by Dysfunction of Wrn

**Figure 1:**
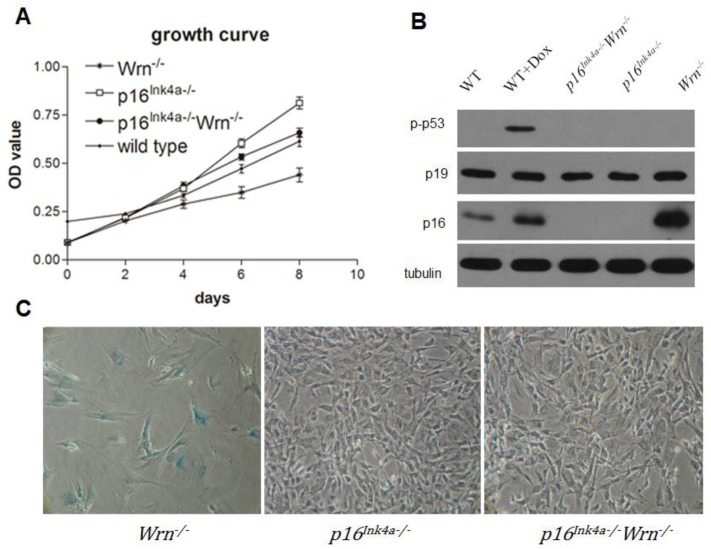
*Wrn^−/−^* induced up-regulation of *p16^Ink4a^* and caused a growth barrier in MEFs. (**A**) Growth curves of wild type, *p16^Ink4a−/−^*, *Wrn^−/−^* and *p16^Ink4a−/−^Wrn^−/−^* MEFs; (**B**) Western blot analysis of *p16^Ink4a−/−^*, *Wrn^−/−^* and *p16^Ink4a−/−^Wrn^−/−^* MEFs. Wild type MEFs (WT) with or without doxorubicin treatment were used as controls. (**C**) SA-β-Gal staining of *p16^Ink4a−/−^*, *Wrn^−/−^* and *p16^Ink4a−/−^Wrn^−/−^* MEFs at passage 13.

To investigate the growth potential of MEFs with the genotypes *p16^Ink4a−/−^*, *Wrn^−/−^*, and *p16^Ink4a−/−^Wrn^−/−^*, we performed a growth curve analysis using wild type MEFs as a control. We found that *Wrn^−/−^* MEFs grew slower than *p16^Ink4a−/−^* MEFs; however, knock out of p16^Ink4a^ function (*p16^Ink4a−/−^Wrn^−/−^*) rescued the growth potential of *Wrn^−/−^* MEFs (Figure 1A). These data suggest that the growth barrier caused by dysfunction of Wrn is overcome by the loss of p16^Ink4a^ function. Furthermore, western blot analysis revealed that compared to wild type MEFs (Figure 1B, WT), *Wrn^−/−^* MEFs have dramatically increased p16^Ink4a^ expression (Figure 1B, *Wrn^−/−^*). This result suggests that loss of Wrn function stimulates the expression of p16^Ink4a^, which might cause the growth barrier of *Wrn^−/−^* MEFs due to negative regulation of the cell cycle. Thus, knockout of p16^Ink4a^ function could overcome the growth barrier caused by a defect in Wrn. Surprisingly, we did not detect an up-regulation of phosphorylated p53 in *Wrn^−/−^* MEFs, suggesting that unlike doxorubicin treatment, the loss of Wrn did not cause sufficient DNA damage to stimulate the p53 pathway (Figure 1B, p-p53). In addition, p19^Arf^, a response protein to oncogenic stimulation, did not show up-regulation in the absence of Wrn (Figure 1B, p19). Furthermore, we performed SA-β-Gal staining to detect cellular senescence in passage 13 of *p16^Ink4a−/−^*, *Wrn^−/−^*, and *p16^Ink4a−/−^Wrn^−/−^* MEFs. The results indicated that cellular senescence occurred in passage 13 of *Wrn^−/−^* MEFs (57.31 ± 7.19% of cells were positive for SA-β-Gal staining), but not in *p16^Ink4a−/−^* MEFs (1.40 ± 0.53% cells were positive for SA-β-Gal staining) or *p16^Ink4a−/−^Wrn^−/−^* MEFs (2.23 ± 1.86% cells were positive for SA-β-Gal staining) (Figure 1C).

### 2.2. DNA Damage Response to Genotoxin or Telomere Dysfunction in Cells Lacking Wrn or p16^Ink4a^ Function

To further understand cellular senescence caused by telomere dysfunction in Werner syndrome and the role of Wrn and p16^Ink4a^ in the DNA damage response to telomere dysfunction, a well defined telomere damaging protein TRF2^ΔBΔM^ [13] was introduced into *p16^Ink4a−/−^*, *Wrn^−/−^* and *p16^Ink4a−/−^Wrn^−/−^* MEFs. The global genotoxin doxorubicin was used in parallel to investigate the distinct stimulation of pathways involved in the DNA damage response.

**Figure 2:**
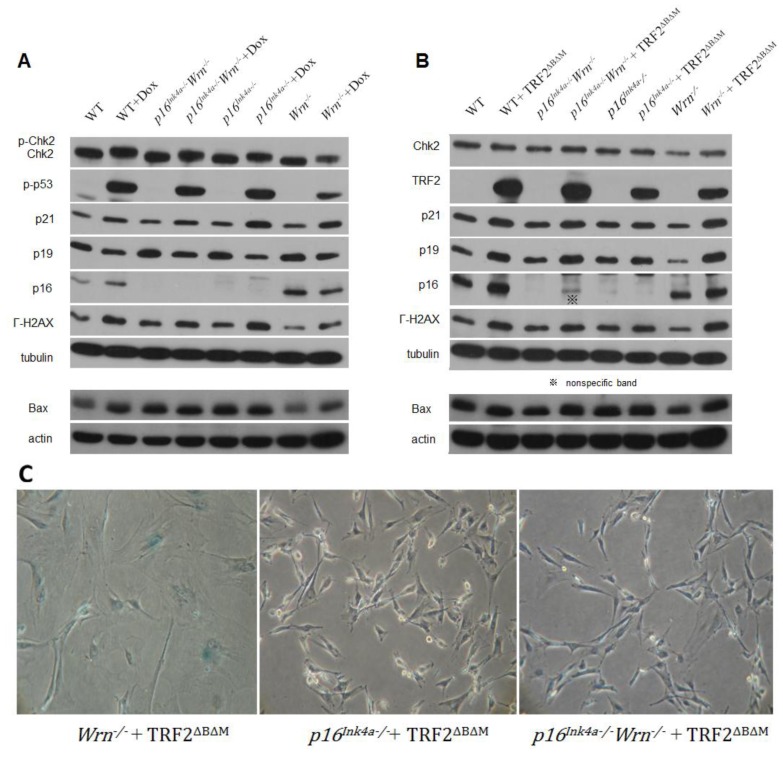
DNA damage response to genotoxin or telomere dysfunction in cells lacking either Wrn or p16^Ink4a^ function. (**A**) Western blot analysis to determine the response of MEFs to doxorubicin treatment; (**B**) Western blot analysis to determine the response of MEFs to TRF2^ΔBΔM^ overexpression. Wild type MEFs (WT) were used as controls; (**C**) SA-β-Gal staining of *p16^Ink4a−/−^*, *Wrn^−/−^* and *p16^Ink4a−/−^Wrn^−/−^* MEFs following TRF2^ΔBΔM^ overexpression.

Western blot analysis demonstrated that in response to the global DNA damage caused by doxorubicin, Chk2, p53, and H2AX proteins were phosphorylated in wild type, *p16^Ink4a−/−^*, *Wrn^−/−^* and *p16^Ink4a−/−^Wrn^−/−^* MEFs (Figure 2A, Chk2, p-p53, γ-H2AX). These data confirm that the regular DNA damage responses were stimulated in MEFs lacking Wrn or p16^Ink4a^ (Figure 2A, *Wrn^−/−^*). Interestingly, in response to telomere dysfunction induced by the overexpression of TRF2^ΔBΔM^ (Figure 2B, TRF2), Chk2 was not phosphorylated (Figure 2B, Chk2). In addition, no phosphorylated p53 could be detected (data not shown), suggesting a lack of response of Chk2-p53 to telomere dysfunction induced by TRF2^ΔBΔM^ in the genetic context of these MEFs. However, p21 proteins were slightly up-regulated despite the p53 phosphorylation status (Figure 2B, p21). This result could be due to a p53-independent activation of p21 or a low level of p53 activation that is not detectable by western blot. H2AX proteins were phosphorylated significantly in response to TRF2^ΔBΔM^ overexpression in wild type, *p16^Ink4a−/−^*, *Wrn^−/−^*, and *p16^Ink4a−/−^Wrn^−/−^* MEFs (Figure 2B, γ-H2AX), suggesting that TRF2^ΔBΔM^ induces the activation of the DNA damage response. p16^Ink4a^ proteins were remarkably up-regulated in response to TRF2^ΔBΔM^ overexpression in wild type MEFs, as well as *Wrn^−/−^* MEFs (Figure 2B, p16). These data suggest that in *p16^Ink4a−/−^* and *p16^Ink4a−/−^Wrn^−/−^* MEFs, the loss of p16^Ink4a^ function prevents cells from undergoing TRF2^ΔBΔM^-induced senescence.

Surprisingly, p19^Arf^ proteins were up-regulated in response to TRF2^ΔBΔM^ overexpression in wild type, *p16^Ink4a−/−^*, *Wrn^−/−^* and *p16^Ink4a−/−^Wrn^−/−^* MEFs (Figure 2B, p19), which was contrary to the response of MEFs to doxorubicin in which p19^Arf^ proteins were slightly down-regulated (Figure 2A, p19). These western blots were quantified (supplementary data 1).

Interestingly, the background expression levels of the apoptotic marker Bax were up-regulated in *p16^Ink4a−/−^* and *p16^Ink4a−/−^Wrn^−/−^* MEFs, but not in *Wrn^−/−^* MEFs. Upon doxorubicin treatment or TRF2^ΔBΔM^ overexpression, Bax was not up-regulated (Figure 2A,B, Bax, actin was used as the loading control). SA-β-Gal staining of *p16^Ink4a−/−^*, *Wrn^−/−^* and *p16^Ink4a−/−^Wrn^−/−^* MEFs after TRF2^ΔBΔM^ overexpression showed that *Wrn^−/−^* MEFs senesced rapidly after TRF2^ΔBΔM^ overexpression, while *p16^Ink4a−/−^Wrn^−/−^* MEFs did not appear to senesce (Figure 2C). These data further support the conclusion that loss of p16^Ink4a^ function prevents cells from TRF2^ΔBΔM^-induced senescence.

Telomere dysfunction could induce chromosomal aberrations, which further enhance the destructiveness of telomere dysfunction, ultimately resulting in tumorigenesis.

To investigate the chromosomal aberrations induced by TRF2^ΔBΔM^ overexpression in *p16^Ink4a−/−^*, *Wrn^−/−^* and *p16^Ink4a−/−^Wrn^−/−^* MEFs, we attempted to harvest chromosomes and perform cytogenetic analysis. However, TRF2^ΔBΔM^ overexpression leads to cellular senescence in wild type and *Wrn^−/−^* MEFs, thereby preventing us from harvesting chromosomes from these cells for analysis. In contrast, despite the overexpression of TRF2^ΔBΔM^, *p16^Ink4a−/−^* and *p16^Ink4a−/−^Wrn^−/−^* MEFs continuously grow. We harvested the chromosomes from early passaged (p4), medium passaged (p18), and late passaged (p27) cells. Chromosome analysis (Table 1) showed that very few chromosomal breakages or fusions occurred in early and medium passages of MEFs, late passages of *p16^Ink4a−/−^Wrn^−/−^* MEFs that were overexpressing TRF2^ΔBΔM^ accumulated chromosomal fusions (12 of 30 cells in metaphase), and only a few fusions were found in late passages of *p16^Ink4a−/−^* MEFs (2 of 30 cells in metaphase). We observed an increase of aneuploidy that correlated with increasing numbers of cell passages in both types of MEFs, which might be due to the cell passaging process itself. These data suggest that loss of p16^Ink4a^ function in a Wrn-deficient background rescues cellular senescence, but with the price of accumulating chromosomal aberrations.

### 2.3. Loss of p16^Ink4a^ Function Facilitated the Elimination of Exogenous TRF2^ΔBΔM^ in MEFs

**Figure 3:**
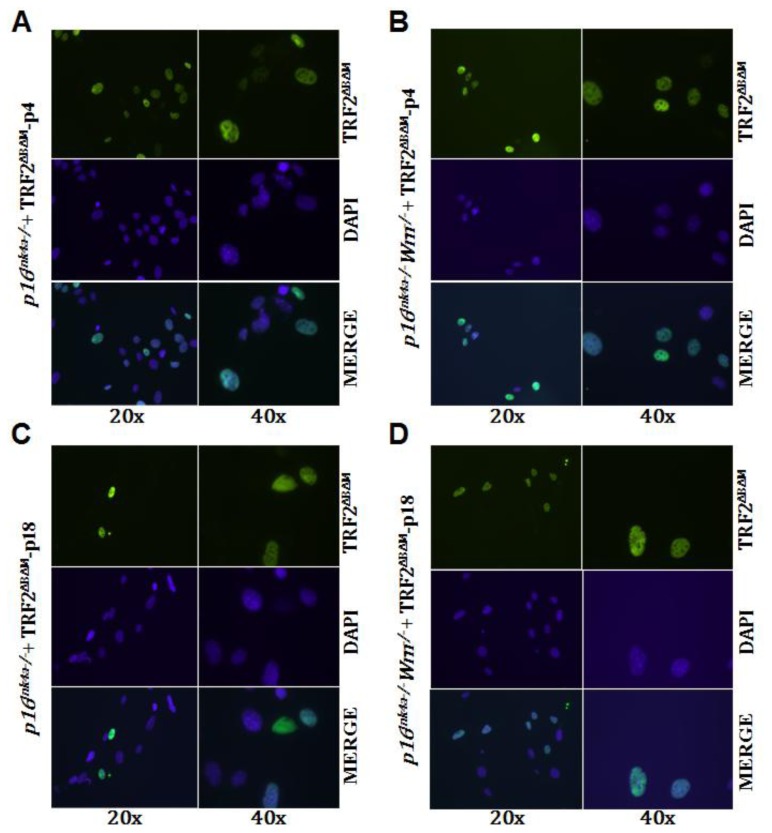
Overexpression of TRF2^ΔBΔM^ in early and medium passages of *p16^Ink4a−/−^* and *p16^Ink4a−/−^Wrn^−/−^* MEFs revealed by immunostaining (20× and 40× refer to the amplification power of the objective lens, DAPI was used to show the nuclear localization). (**A**) Early passage (P4) of *p16^Ink4a−/−^* MEFs; (**B**) Early passage (P4) of *p16^Ink4a−/−^Wrn^−/−^* MEFs; (**C**) Late passage (P18) of *p16^Ink4a−/−^* MEFs; (**D**) Late passage (P18) of *p16^Ink4a−/−^Wrn^−/−^* MEFs.

The above data raise the question of how *p16^Ink4a−/−^* and *p16^Ink4a−/−^Wrn^−/−^* MEFs could continuously grow while maintaining the overexpression of TRF2^ΔBΔM^. First, we confirmed the overexpression of TRF2^ΔBΔM^ in early and medium passages of *p16^Ink4a−/−^* and *p16^Ink4a−/−^Wrn^−/−^* MEFs using immunostaining. As expected, we could detect expression of TRF2^ΔBΔM^ in the nuclei of early passages (p4) of *p16^Ink4a−/−^* and *p16^Ink4a−/−^Wrn^−/−^* MEFs (Figure 3A,B). However, in passage 18 of *p16^Ink4a−/−^* and *p16^Ink4a−/−^Wrn^−/−^* MEFs, the number of cells with positive staining for TRF2^ΔBΔM^ decreased dramatically (Figure 3C,D).

**Figure 4:**
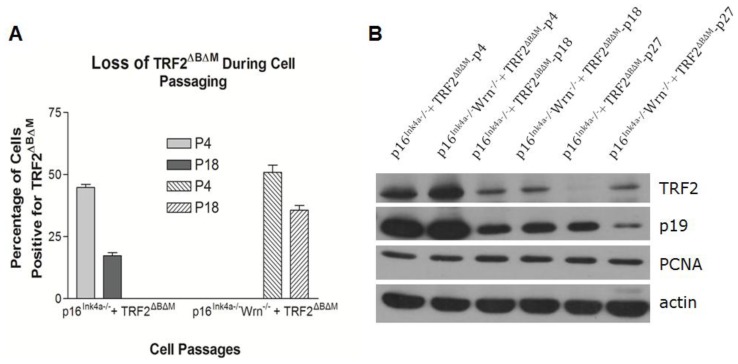
Elimination of TRF2^ΔBΔM^ expression in the late passages of *p16^Ink4a−/−^* and *p16^Ink4a−/−^Wrn^−/−^* MEFs. (**A**) The percentage of cells positive for TRF2^ΔBΔM^ measured by immunostaining; (**B**) Western blot showing the decrease of TRF2^ΔBΔM^ expression with increasing cell passages.

The percentage of P4 *p16^Ink4a−/−^* MEFs with positive staining for TRF2^ΔBΔM^ was around 45%, while positive staining of P18 *p16^Ink4a−/−^* MEFs decreased to 20% (Figure 4A). Similarly, positive staining of P4 *p16^Ink4a−/−^Wrn^−/−^* MEFs was around 56%, while positive staining of P18 *p16^Ink4a−/−^Wrn^−/−^* MEFs decreased to 39% (Figure 4A). Western blotting further confirmed the decrease of TRF2^ΔBΔM^ expression in MEFs with the increasing cell passage number (Figure 4B, supplementary data 2). These data suggest that in the absence of p16^Ink4a^, MEFs are able to lose expression of telomere dysfunction factors and, therefore, bypass senescence and sustain cell growth.

When the mouse model of Werner Syndrome (*Wrn^−/−^*) was established by deleting a segment of the Wrn gene, the premature aging phenotype of human Werner Syndrome was not observed [23]. It is now clear that this observation was due to the endogenous telomerase activity in mouse somatic cells [19,20]. While *Wrn^−/−^* mice display reduced embryonic survival, live-born mice appear normal during their first year of life. However, *Wrn^−/−^* MEFs display a prematurely reduced growth rate as a function of passage number in culture, demonstrating that *Wrn^−/−^* MEFs grow more slowly and have a lower saturation density in culture than their heterozygous and wild type counterparts [23].

Using *Wrn^−/−^* MEFs, we also observed the low growth potential of *Wrn^−/−^* MEFs, which may be due to the up-regulation of the cell cycle inhibitor p16^Ink4a^. Knockout of p16^Ink4a^ rescued the growth potential of *Wrn^−/−^* MEFs, further supporting this point.

Furthermore, the loss of either Wrn or p16^Ink4a^ function did not dramatically affect the cellular DNA damage response to the global genotoxin doxorubicin. However, when the dominant negative inhibitor of TRF2 (TRF2^ΔBΔM^) was introduced into the cells and induced cellular senescence, the up-regulation response of p16^Ink4a^ was disabled in p16^Ink4a^-deficient cells, which resulted in distinct cellular consequences between *Wrn^−/−^* MEFs and *p16^Ink4a−/−^Wrn^−/−^* MEFs. In addition, the up-regulation of γ-H2AX in response to TRF2^ΔBΔM^ is reduced in *p16^Ink4a−/−^Wrn^−/−^* MEFs. Together these regulations in *p16^Ink4a−/−^Wrn^−/−^* MEFs overcame the cellular senescence induced by telomere dysfunction and sustained the continuous growth of cells with the consequence of the accumulation of chromosomal aberrations. Eventually *p16^Ink4a−/−^Wrn^−/−^* MEFs and *p16^Ink4a−/−^* MEFs could lose expression of TRF2^ΔBΔM^, which might further stabilize the chromosomes and stop the accumulation of chromosomal aberrations. However, we found that in *p16^Ink4a−/−^Wrn^−/−^* MEFs the chromosomal aberrations still accumulated slowly due to the existing overexpression of TRF2^ΔBΔM^. In this case, the reduction of TRF2^ΔBΔM^ levels slowed the accumulation of chromosomal aberrations. The mechanism responsible for the loss of TRF2^ΔBΔM^ expression is not clear; however, the loss of p16^Ink4a^ function played a permissive role in this process because the *Wrn^−/−^* MEFs could not survive TRF2^ΔBΔM^ overexpression. In contrast to a previous report [24], we did not observe p53 activation in response to TRF2^ΔBΔM^ overexpression in these MEFs. A previous study also indicated that in normal human epidermal keratinocytes, TRF2^ΔBΔM^ overexpression caused only a two fold increase in both the phosphorylation of p53 at serine 15 and 53BP1 DNA damage foci and no detectable increase in p21. Despite the weak DNA damage response, the keratinocytes demonstrated growth arrest, reduced colony formation and senescence [25]. Another study demonstrated that uncapping telomeres with TRF2^ΔBΔM^ resulted in apoptosis, not senescence. These authors found a more dramatic activation of p53, as well as an up-regulation of Bax [14]. In our study, however, we did not observe activation of Chk2 or p53 or up-regulation of Bax. It is possible that a higher level of TRF2^ΔBΔM^ results in apoptosis rather than senescence. It has also been shown that in human fibroblasts, telomere dysfunction induced senescence and signaled through ATM-p53-p21, but not p16^Ink4a^, suggesting that distinct senescence programs could progress in parallel, resulting in mosaic cultures as well as individual cells responding to multiple signals [26].

Together, these data suggest different roles of p53 and p16^Ink4a^ in response to distinct DNA damages and shed light on anti-aging strategies by the regulation p16^Ink4a^ expression.

## 3. Experimental Section

### 3.1. Cell Lines, Constructs and Antibodies

MEFs were prepared and harvested from individual day 13.5 mouse embryos from designed parental mice with *p16^Ink4+/−^* or *Wrn^+/−^* genotypes (heterozygous knockouts of either *p16^Ink4^* or *Wrn*) and the harvested MEFs were cultured and genotyped. MEFs with wild type, *p16^Ink4a−/−^*, *Wrn^−/−^* or *p16^Ink4a−/−^Wrn^−/−^* genotypes were used for experiments. Wild type MEFs were used for experiments before passage 10 and the cells were passaged at a ratio of 1:2 or 1:3 so that the cells were not excessively diluted and senesced too quickly.

All cell lines were cultured in DMEM medium supplemented with 10% fetal bovine serum (Hyclone, CA, USA) in a 3% oxygen and 5% CO_2_ incubator at 37 °C.

The pBabe-TRF2^ΔBΔM^ (with a cMyc tag) construct, a gift from Dr. de Lange’s lab (as described in [27]), was introduced into MEFs. For transient transfection, cells were used 48 h after transfection. For stable expression, antibiotic selection was applied for 1–2 months and expression of TRF2^ΔBΔM^ was analyzed by both western blot and immunostaining.

Antibodies used for western blot were anti-p16^Ink4a^ (M-156) (1:500, Santa Cruz, CA, USA), anti-Chk2 (1:500, BD Transduction Laboratories, CA, USA), anti-phospho-p53 (Ser15) (1:500, Cell Signaling, MA, USA), anti-cMyc (9E10) (1:500, Santa Cruz, CA, USA), anti-p19^Arf^ (1:1250, Upstate, NY, USA), anti-γ-H2AX (1:1000, Upstate, NY, USA), and anti-γ-tubulin (1:5000, Upstate, NY, USA).

### 3.2. Growth Curve Assay

A total of 2.5 × 10^4^ cells were seeded per well in 12 well plates and triplicates were prepared for each sample. At each time point, cells were fixed for 20 min at room temperature in 10% buffered formalin and stained with 1 mL 0.1% crystal violet for 20 min at room temperature. After the plates were washed and dried, cells were treated with 2 mL 10% acetic acid and the OD of the extracted dye was measured at 595 nm. Statistical analysis was performed to compare the difference between cell lines (*n* = 3).

### 3.3. Immunostaining Assay

Cells were fixed with 2% paraformaldehyde and 2% sucrose in 1× PBS (pH 7.6) for 10 min and then permeabilized with 1% NP-40. After pre-incubation with 5% BSA/PBS, cells were incubated first with the primary antibody and then with the secondary antibody in 1% BSA/PBS for 1 h at room temperature. After DAPI staining, the slides were mounted with ProLong mounting medium (Invitrogen, CA, USA). Fluorescence microscopy was performed using a Nikon Eclipse 90i fluorescence microscope and a Nikon Digital Sight CCD (controlled by NIS-Elements 3.0 software).

### 3.4. SA-β-Gal Staining

SA-β-Gal staining was performed as described previously [28]. Briefly, cultured cells were washed in 1× PBS and fixed for 3–5 min (room temperature) in 2% formaldehyde, 0.2% glutaraldehyde. Fixed cells were stained with fresh stain solution for SA-β-galactosidase activity at 37 °C for 4 h. The percentage of cells positive for SA-β-Gal staining were quantified and statistically analyzed (*n* = 3).

## 4. Conclusions

Our data suggest that, in the context of Wrn deficiency-related telomere dysfunction, loss of p16^Ink4a^ function could prevent cells from senescence. These results shed light on the anti-aging strategy by regulation of p16^Ink4a^ expression.

## Supplementary Information



## Figures and Tables

**Table 1:** Chromosome Analysis in MEFs overexpressing TRF2^ΔBΔM^.

MEFs’ Genotype	metaphases counted	breakage	fusion	Diploid 2*N* = 40	Aneuploid 2*N* > 40	Aneuploid 2*N* < 40
*p16^Ink4a−/−^* + TRF2^ΔBΔM^-p4	30		1	15	14	
*p16^Ink4a−/−^Wrn^−/−^* + TRF2^ΔBΔM^-p4	30			14	14	2
*p16^Ink4a−/−^* + TRF2^ΔBΔM^-p18	30	1		7	20	2
*p16^Ink4a−/−^Wrn^−/−^* + TRF2^ΔBΔM^-p18	30			15	13	2
*p16^Ink4a−/−^* + TRF2^ΔBΔM^-p27	30		2	1	27	
*p16^Ink4a−/−^Wrn^−/−^* + TRF2^ΔBΔM^-p27	30		12	1	16	
