# Supplementary material for: Loss of p16Ink4a Function Rescues Cellular Senescence Induced by Telomere Dysfunction
Source: Int J Mol Sci. 2012 May 16;13(5):5866–77. doi: 10.3390/ijms13055866 (PMC3382785; doi:10.3390/ijms13055866)
Supplement: Supplementary file 1 [file ijms-13-05866-s001.pdf]

Supplementary Information

Figure S1. The quantification of Figure 2A and B. The blots were quantified based on their loading control tubulin or actin. The density of blots for WT cells were set as 1.

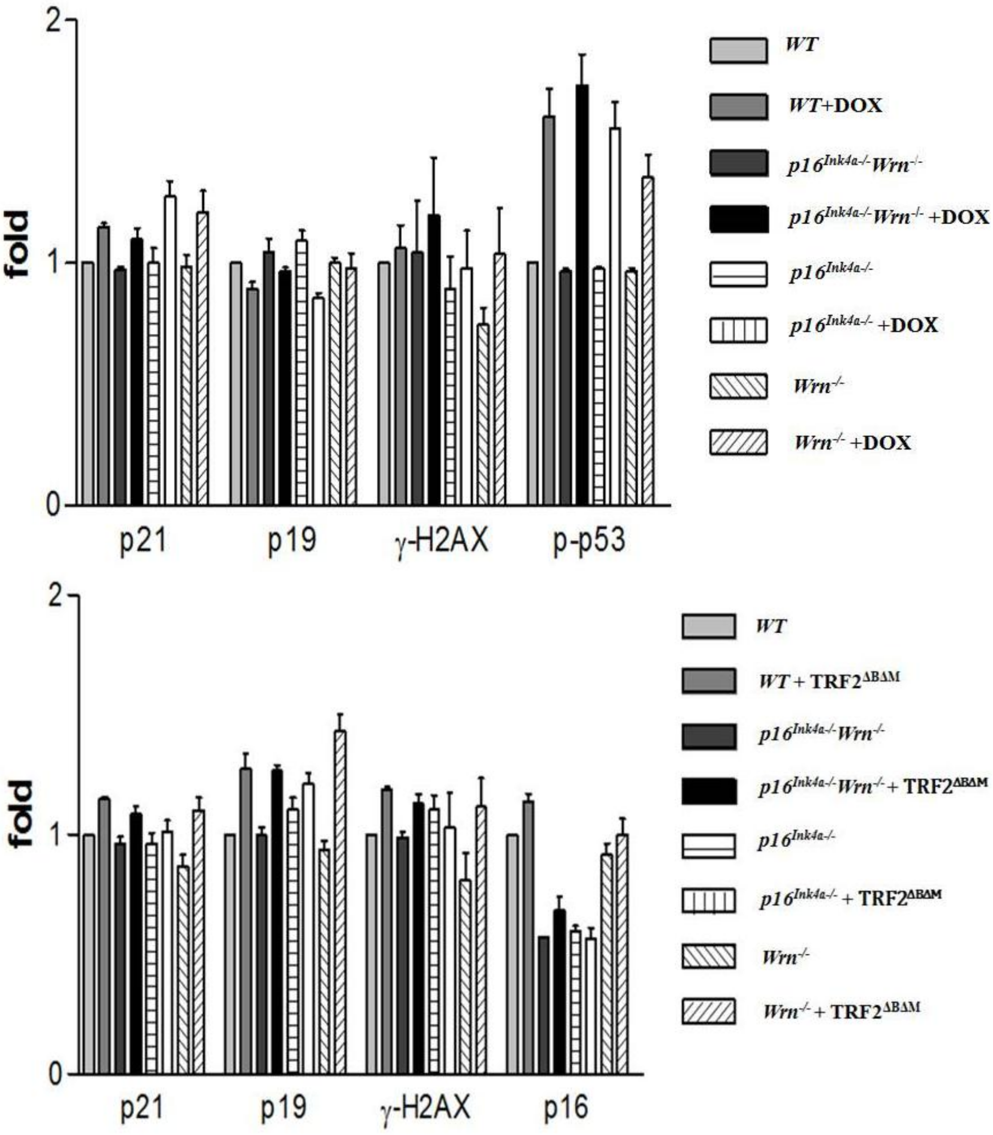

Figure S2. The quantification of Figure 4B. The blots were quantified based on their loading control tubulin or actin. The density of blots for WT cells were set as 1.

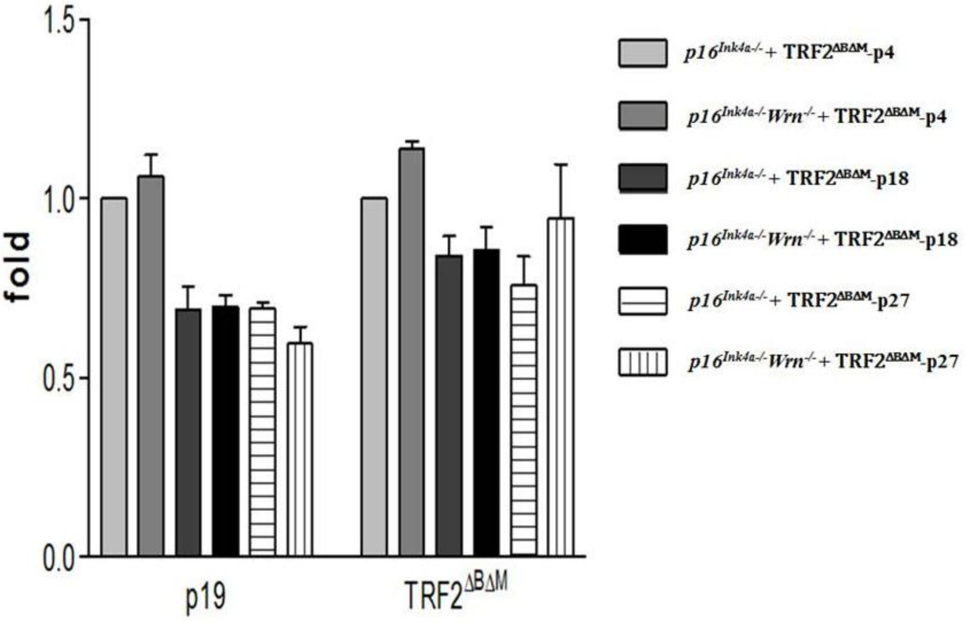

© 2012 by the authors; licensee MDPI, Basel, Switzerland. This article is an open access article distributed under the terms and conditions of the Creative Commons Attribution license (http://creativecommons.org/licenses/by/3.0/).
